# GPU-Accelerated Molecular Dynamics Simulation to Study Liquid Crystal Phase Transition Using Coarse-Grained Gay-Berne Anisotropic Potential

**DOI:** 10.1371/journal.pone.0151704

**Published:** 2016-03-17

**Authors:** Wenduo Chen, Youliang Zhu, Fengchao Cui, Lunyang Liu, Zhaoyan Sun, Jizhong Chen, Yunqi Li

**Affiliations:** 1 Key Laboratory of Synthetic Rubber & Laboratory of Advanced Power Sources, Changchun Institute of Applied Chemistry, Chinese Academy of Sciences, 5625 Renmin Street, Changchun, PR China; 2 State Key Laboratory of Polymer Physics and Chemistry, Changchun Institute of Applied Chemistry, Chinese Academy of Sciences, 5625 Renmin Street, Changchun, PR China; Hong Kong University of Science and Technology, HONG KONG

## Abstract

Gay-Berne (GB) potential is regarded as an accurate model in the simulation of anisotropic particles, especially for liquid crystal (LC) mesogens. However, its computational complexity leads to an extremely time-consuming process for large systems. Here, we developed a GPU-accelerated molecular dynamics (MD) simulation with coarse-grained GB potential implemented in GALAMOST package to investigate the LC phase transitions for mesogens in small molecules, main-chain or side-chain polymers. For identical mesogens in three different molecules, on cooling from fully isotropic melts, the small molecules form a single-domain smectic-B phase, while the main-chain LC polymers prefer a single-domain nematic phase as a result of connective restraints in neighboring mesogens. The phase transition of side-chain LC polymers undergoes a two-step process: nucleation of nematic islands and formation of multi-domain nematic texture. The particular behavior originates in the fact that the rotational orientation of the mesogenes is hindered by the polymer backbones. Both the global distribution and the local orientation of mesogens are critical for the phase transition of anisotropic particles. Furthermore, compared with the MD simulation in LAMMPS, our GPU-accelerated code is about 4 times faster than the GPU version of LAMMPS and at least 200 times faster than the CPU version of LAMMPS. This study clearly shows that GPU-accelerated MD simulation with GB potential in GALAMOST can efficiently handle systems with anisotropic particles and interactions, and accurately explore phase differences originated from molecular structures.

## 1. Introduction

Liquid crystals (LC) can be assembled from either small molecules [1, 2] or polymers [3–6]. Compared with small molecular LC, liquid crystal polymers (LCP) offer both mesogen orientation and polymer mechanical merit [7, 8]. The orientation of mesogens competing the penalty from the conformational entropy leads to abundant phase behaviors [9–11]. Nowadays, computer simulations have been widely used to improve our understanding of these phase behaviors which are not fully accessible by experimental approaches [12–14]. Adequate descriptions of the LC systems require an accurate model for both inter- and intra-molecular interactions, and also require system sizes that are large enough to observe the effects of molecular ordering [15]. A combination of coarse-grained Gay-Berne (GB) model [16–18] and graphics processing units (GPU)-accelerated algorithms [19–22] should be a highly promising way to provide simulation accuracy and efficiency in the study of larger systems considering more details of anisotropic particles, especially for LC phase transitions.

Coarse graining as one of the conceptual and technical ways that smoothes over or averages out some of fine details, has been widely used to simulate the longer time- and larger length-scale dynamics and phase behaviors [23, 24]. The coarse graining of LC through representing groups of atoms by single interaction sites leads to a remarkable acceleration in molecular simulation. Conventionally, LC mesogens are coarse grained as spheres in a wireframe graph, such as Kihara model [25], multisite model [13], and Lennard-Jones (LJ) model [26]. The GB potential employs soft ellipsoids rather than spherical particles to model particles with anisotropic characteristics [16, 17], typically like discotic metallomesogens and dendrimers etc [27–29]. The interaction and the corresponding geometric packing of ellipsoidal particles have four forms, i.e., side-to-side, end-to-end, cross and T-shape. Through the consideration of anisotropic attraction and repulsion, the GB potential has been successfully used to model thermotropic LC phase transition for mesogens in small molecules [30–34]. De Miguel *et al*. identified the isotropic, nematic and smectic-B phases in small molecular LC phases and found that the stability of nematic phase is strongly influenced by the anisotropy in the well-depth of GB potential [35, 36]. Luckhurst and his coworkers found that the stability of the isotropic and nematic phases is dominated by short range anisotropic repulsive forces, while the stability of the smectic-A phase is dominated by the anisotropic attractive forces in GB potential[37].

According to the location of mesogens in backbone or in side-chain pendant, LCP are classified into main-chain liquid crystal polymers (MCLCP) and side-chain liquid crystal polymers (SCLCP) [38]. The competition between the enthalpy gain from the global packing of mesogens and the entropy penalty from the conformation of polymer backbones and spacers leads to complicate phase behaviors which have attracted intensive studies [3, 39, 40]. Wilson *et al*. pioneered the GB/LJ model and offered a clear statement on the influence of odd-even spacer lengths on the LC phase transition in MCLCP [3, 41, 42]. In the GB/LJ model, rigid units are modeled by ellipsoidal particles described by GB potential, and flexible spacers are described by conventional LJ spherical particles. Allen *et al*. applied the GB/LJ model and proposed the mechanism for the thermotropic isotropic-LC phase transition of polyester with temperature decreasing [3]. Compared with well studied MCLCP, for SCLCP, the structure of polymer backbone, the grafting density, the pendant mesogens, and the flexible spacer length all exert important influence on the mesophase morphology [43]. SCLCP with highly flexible backbones (e.g., methacrylate based) and longer spacers have a lamellar smectic phase, while the same polymers with a short spacer typically have the nematic and isotropic phases at high temperatures [34]. Experimentally, neutron scattering[44], X-Ray Scattering[45] and birefringence measurements[46, 47] have been steadily revealed abundant morphologies originated from the assembly of molecules with different mesogens and architectures. How to accurately address the relationship between the morphology of LC phase and the molecular structures is still of key importance.

Although GB potential has been wide accepted as an accurate model for LC mesogens, the computational complexity aroused challenges in simulations to identify LC phase transition in sufficient temporal and spatial scales. Various efforts including the simplification of energy function, the utilization of parallel computation or GPU-acceleration have been paid to reduce computational cost for GB potential computation. Zannoni and coworkers proposed a simplification by a sum of stretching and bending potentials for adjacent GB mesogens which can significantly reduce the computational cost [48, 49]. A modified GB potential proposed by Persson can save 10% to 20% computational cost with reasonable approximation compared to the original GB potential [50]. Alternatively, Wilson *et al*. developed parallel MD simulation algorithms named GBMOL and GBMOL DD [39] to enlarge simulation systems. They realized a simulation with more 40960 atoms and observed novel patterns of LC phases. Meanwhile, the utilization of GPU acceleration enables MD simulation to study complicate phase behaviors of polymers within acceptable computational time [51–54]. Sunarso *et al*. used a GPU-accelerated MD simulation to study the generation of macroscopic flow through molecular reorientation in nematic LC phase which is triggered by an electric field [53]. They found that the GPU-acceleration can provide about 50 times faster than that using a single CPU core on an identical simulation. A series of software packages utilizing GPU-acceleration in generalized MD simulation have been developed, such as LAMMPS-GPU [55], HOOMD-blue [56] and GALAMOST [57]. Among them, GALAMOST fully took advantages of the mesoscopic simulation techniques including the soft anisotropic particle model [58], the iterative Boltzmann inversion numerical potential coarse-graining method [59], the hybrid particle-field MD [60], and the chain-growth polymerization model [61] etc. It has shown great potentials to efficiently model various nanoparticles [62–64] and polymeric materials [65, 66].

In this study, we developed GPU-accelerated MD simulations equipped with coarse-grained GB potential implemented in GALAMOST package to investigate the LC phase transitions. The model and simulation details are presented in Section 2. Computation cost is compared in the simulation of mesogens in small molecules in Section 3. The difference in phase morphologies originated from mesogens in small molecular LC, MCLCP and SCLCP are discussed in Section 4.

## 2. Model and Simulation

### 2.1 Gay-Berne Potential

Generally, the interaction between spherical beads can be calculated by a LJ potential, *U*_*LJ*_

$$U_{LJ}\;=\;4\varepsilon_{0}[{\left(\frac{\sigma_{0}}{r_{ij}}\right)}^{12}\;-\;{\left(\frac{\sigma_{0}}{r_{ij}}\right)}^{6}]\;$$ (1)

where *r*_*ij*_ is the distance between beads *i* and *j* located at **r**_*i*_ and **r**_*j*_. The parameters *ε*_0_ and *σ*_0_ are taken as the units of energy and length, respectively.

Then the GB potential is defined using an analogous form of the LJ potential

$$U({\hat{u}}_{i},{\hat{u}}_{j},r_{ij})=4\varepsilon_{0}\varepsilon ({\hat{u}}_{i},{\hat{u}}_{j},{\hat{r}}_{ij})\times \left\{\;{\left[\frac{\sigma_{0}}{r_{ij}-\sigma ({\hat{u}}_{i},{\hat{u}}_{j},{\hat{r}}_{ij})+\sigma_{0}}\right]}^{12}-{\left[\frac{\sigma_{0}}{r_{ij}-\sigma ({\hat{u}}_{i},{\hat{u}}_{j},{\hat{r}}_{ij})+\sigma_{0}}\right]}^{6}\;\right\}$$ (2)

**Fig 1:**
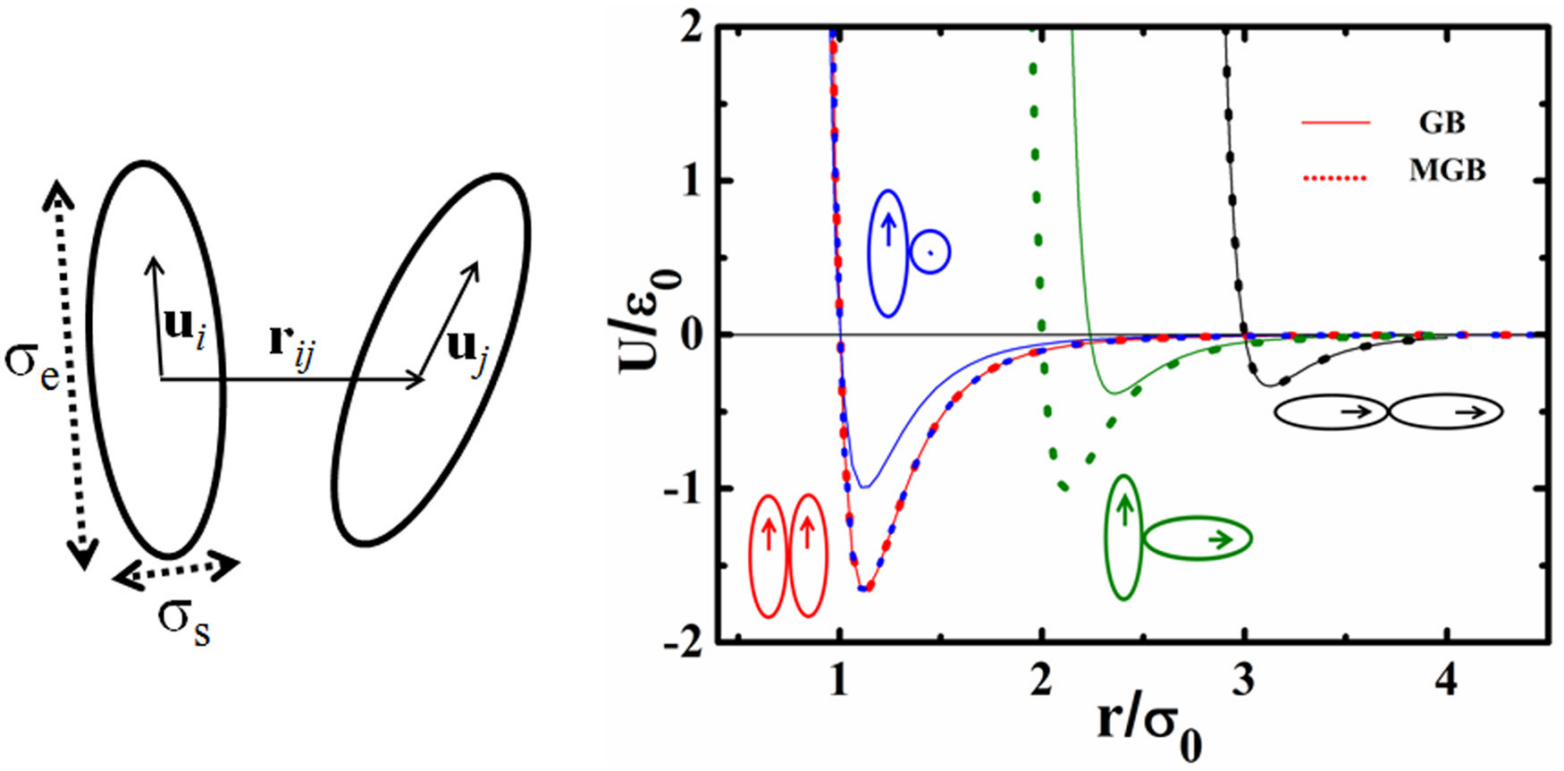
The vectors and parameters of two interacted ellipsoids (left), the interaction profile of GB potential (solid lines) and its modified form (MGB, dotted lines). Interaction profiles in four orientations including side-by-side, cross, T-shape and end-to-end from left to right are presented.

The vector and parameter in the definition are depicted in Fig 1. Here ${\hat{r}}_{ij}$ is the unit vector of **r**_*ij*_ and **û**_*i*_ is the principal vector of an ellipsoidal particle *i*. Functions $\varepsilon ({\hat{u}}_{i},{\hat{u}}_{j},{\hat{r}}_{ij})$ and $\sigma ({\hat{u}}_{i},{\hat{u}}_{j},{\hat{r}}_{ij})$ describe the interaction well-depth and the shape of two ellipsoids. If set one GB ellipsoid to present a typical calamitic mesogen, *σ*_0_ is about 0.5Å and *ε*_0_ is 1.38×10^-21^J.

The shape function is defined as

$$\sigma ({\hat{u}}_{i},{\hat{u}}_{j},{\hat{r}}_{ij})=\sigma_{0}{\left\{1-\frac{\chi }{2}\left[\frac{{\left({\hat{r}}_{ij}\cdot {\hat{u}}_{i}+{\hat{r}}_{ij}\cdot {\hat{u}}_{j}\right)}^{2}}{1+\chi ({\hat{u}}_{i}\cdot {\hat{u}}_{j})}+\frac{{\left({\hat{r}}_{ij}\cdot {\hat{u}}_{i}-{\hat{r}}_{ij}\cdot {\hat{u}}_{j}\right)}^{2}}{1-\chi ({\hat{u}}_{i}\cdot {\hat{u}}_{j})}\right]\right\}}^{-1/2}$$ (3)

where the shape anisotropy parameter *χ* = (*κ*^2^–1)/(*κ*^2^+1) with the geometric aspect ratio *κ* = *σ*_e_ / *σ*_s_. Here the *σ*_e_ is the diameter of the major axis and the *σ*_s_ (= *σ*_0_) is the diameter of the minor axis of an ellipsoid.

The interaction function $\varepsilon ({\hat{u}}_{i},{\hat{u}}_{j},{\hat{r}}_{ij})$ is decoupled into

$$\varepsilon ({\hat{u}}_{i},{\hat{u}}_{j},{\hat{r}}_{ij})=\varepsilon^{\nu }({\hat{u}}_{i},{\hat{u}}_{j})\varepsilon \prime^{\mu }({\hat{u}}_{i},{\hat{u}}_{j},{\hat{r}}_{ij})$$ (4)

with

$$\varepsilon^{\nu }({\hat{u}}_{i},{\hat{u}}_{j})={\left(1-\chi^{2}{\left({\hat{u}}_{i}\cdot {\hat{u}}_{j}\right)}^{2}\right)}^{-1/2\nu }$$ (5a)

and

$$\varepsilon \prime^{\mu }({\hat{u}}_{i},{\hat{u}}_{j},{\hat{r}}_{ij})={\left\{1-\frac{\chi \prime }{2}\left[\frac{{\left({\hat{r}}_{ij}\cdot {\hat{u}}_{i}+{\hat{r}}_{ij}\cdot {\hat{u}}_{j}\right)}^{2}}{1+\chi \prime ({\hat{u}}_{i}\cdot {\hat{u}}_{j})}+\frac{{\left({\hat{r}}_{ij}\cdot {\hat{u}}_{i}-{\hat{r}}_{ij}\cdot {\hat{u}}_{j}\right)}^{2}}{1-\chi \prime ({\hat{u}}_{i}\cdot {\hat{u}}_{j})}\right]\right\}}^{\mu }$$ (5b)

where the interaction enhanced anisotropy parameter *χ'* is defined as $\chi \prime =(k\prime^{1/\mu }-1)/(k\prime^{1/\mu }+1)$. Here *κ'* = *ε*_s_ / *ε*_e_ represents the anisotropy of interactions and *ε*_s_ and *ε*_e_ are the well depths for the side-by-side and the end-to-end configurations, respectively. The empirical tuning exponents *ν* and *μ* are originally set to the values 1 and 2, respectively [3, 16].

In order to reduce the computational complexity of GB potential, Persson introduced a modified GB (MGB) interaction [50]. Both the interaction and the shape functions are simply calculated by

$$\sigma ({\hat{u}}_{i},{\hat{u}}_{j},{\hat{r}}_{ij})=\sigma_{0}[1+\frac{k-1}{2}(\left|{\hat{r}}_{ij}\cdot {\hat{u}}_{i}\right|+\left|{\hat{r}}_{ij}\cdot {\hat{u}}_{j}\right|)]$$ (6a)

$$\varepsilon ({\hat{u}}_{i},{\hat{u}}_{j},{\hat{r}}_{ij})=1+\frac{k\prime^{-1}-1}{2}(\left|{\hat{r}}_{ij}\cdot {\hat{u}}_{i}\right|+\left|{\hat{r}}_{ij}\cdot {\hat{u}}_{j}\right|)$$ (6b)

The interaction profiles of GB and MGB in four typical orientations are shown in Fig 1. The MGB has degenerate state profiles for side-by-side and cross interactions.

### 2.2 Coarse Grained Model

**Fig 2:**
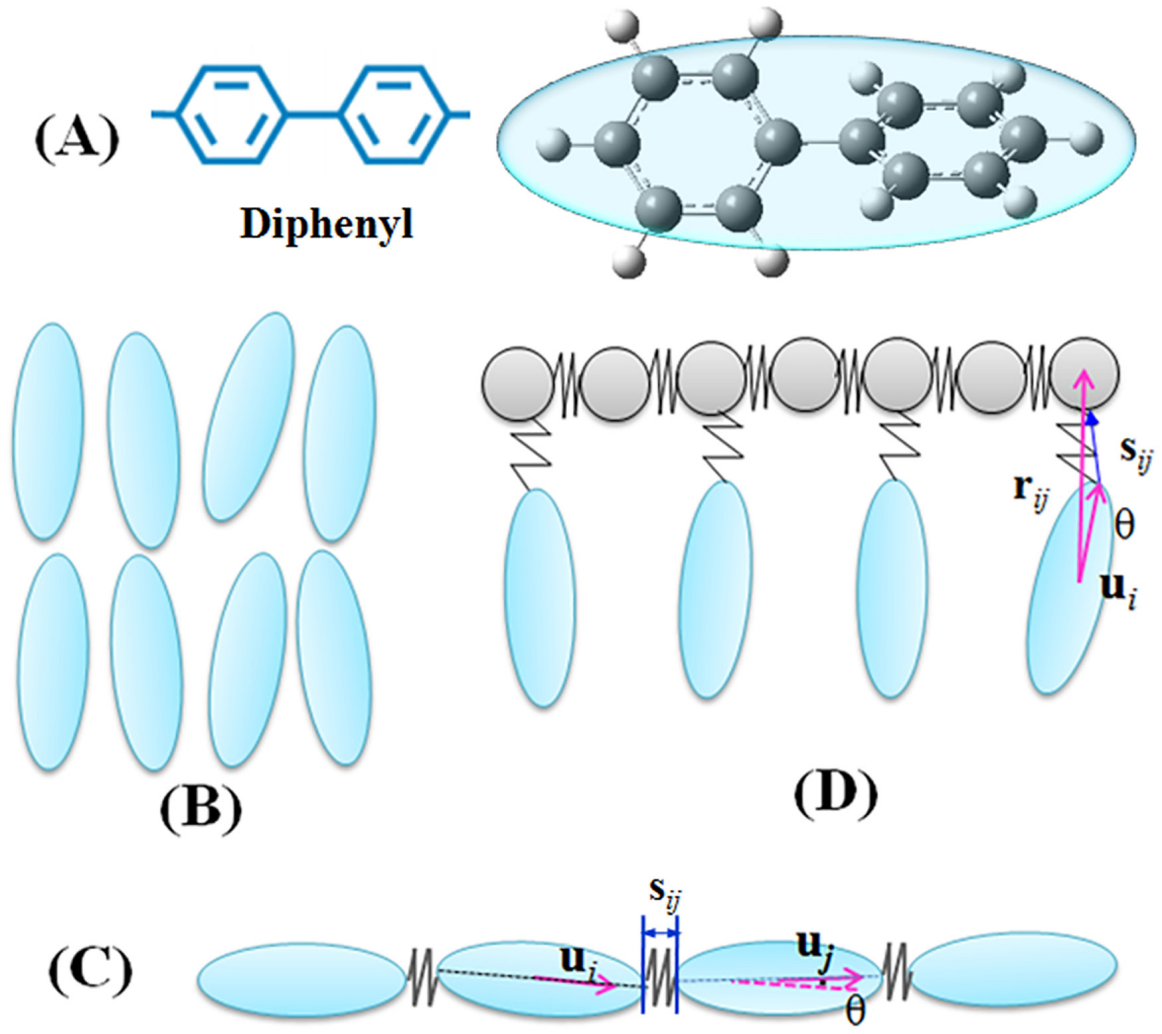
Schematics of LC molecules (A) in small molecular LC (SMALL, B), main-chain polymers (MCLCP, C) and side-chain polymers (SCLCP, D). Ellipsoids are mesogens, spheres are backbone connectors in SCLCP and the springs demonstrate harmonic interactions. *θ* is the angle between the major axis of the two adjacent GB mesogens (**u**_*i*_, **u**_*j*_) for MCLCP (C), while it is the angle between the major axis of the GB mesogen (**u**_*i*_) and the vector from the center of the GB mesogens to the adjacent LJ beads on backbone (**r**_*ij*_) for SCLCP (D). The site-site vector **S**_*ij*_ connects the two adjacent GB/GB sites or GB/LJ sites placed in terminal position for MCLCP and SCLCP, respectively.

For the sake of a reliable comparison on the efficiency of GPU-accelerated MD simulation, we adopted a classical coarse grained model for mesogens proposed by de Miguel *et al*., where the temperature induced LC phase transition of small molecules has been clearly addressed [36]. The mesogens in small molecules, MCLCP and SCLCP are identical. It has a geometric aspect ratio *κ* = 3 and an interaction aspect ratio of *κ'* = 5. The potential is truncated at a distance *r*_cut_ = (*κ* + 1)*σ*_0_ and shifted the interaction at *r*_cut_ to zero. An individual mesogen is treated as a linear rotor, and the moment of inertia around the principal axis of an ellipsoid is I* = *I* / (*mσ*_0_^2^) = 1.0 with *m* = 1.0. Fig 2(A) shows an example like diphenyl and Fig 2(B) presents the small molecular system.

For MCLCP, each mesogen incorporated within the backbone is replaced by a uniaxial ellipsoid, and the adjacent mesogens are bonded by a sum of stretching and bending potentials to maintain molecular topological structures, as shown in Fig 2(C). This model has been used to yield the principal thermotropic LC phases [67]. For SCLCP, the main chain is connected by spherical beads (diameter of *σ*_0_) and a flexible spacer. A harmonic interaction is applied to confine the length and the angle of a spacer while it does not occupy any volume. The parameters describing the GB/LJ interactions are obtained using the venerable Lorentz-Berthelot mixing rules [68]. We use a value *σ*_e_ = 2.0 for the end-to-end manner, *σ*_s_ = 1.0 for the side-by-side manner, *ε*_s_ = 1.29 for the maximum well depth of the GB/LJ interaction and *ε*_e_ = 0.58 for the minimum well depth of the GB/LJ interaction, which is the geometric mean of the GB and the LJ well depths [Fig 2(D)].

Based on this coarse-grained model, the energy function to guide MD simulation is written as

$$\begin{array}{l} U_{\text{total}}=\;\sum_{i=1}^{N_{\text{LJ}}}\sum_{j>i}^{N_{\text{LJ}}}U_{ij}^{\text{LJ}}\text{+}\sum_{i=1}^{N_{\text{GB}}}\sum_{j>i}^{N_{\text{GB}}}U_{ij}^{\text{GB}}+\sum_{i=1}^{N_{\text{GB}}}\sum_{j=1}^{N_{\text{LJ}}}U_{ij}^{\text{GB/LJ}}\\ \;\;\;+\sum_{i=1}^{N_{\text{bond}}}k_{\text{0}}{\text{(}r-r_{\text{0}}\text{)}}^{\text{2}}+\sum_{i=1}^{N_{\text{angle}}}\frac{k_{\text{angle}}}{2}{\left(\text{cos}\theta -\text{cos}\theta_{0}\right)}^{\text{2}} \end{array}$$ (7)

where *N*_LJ_ and *N*_GB_ are number of LJ particles and GB particles, respectively. *U*_*ij*_^LJ^, *U*_*ij*_^GB^ and *U*_*ij*_^GB/LJ^ represent the nonbonded interaction potential. The harmonic interactions of bond and angle are used to model the connectivity between neighboring beads and the rigidity of polymer chains. *N*_bond_ is the number of bonds, *k*_0_ the spring constant, and *r*_0_ the equilibrium bond length of flexible spacer, *r* is the bond length equal to the length of the site-site vector **S**_*ij*_. Chain rigidities are described by a bond bending potential between two consecutive bonds. *N*_angle_ is the number of angles, *k*_angle_ is the bending constant, *θ* is the bond angle and *θ*_0_ the equilibrium bond angle. The spring constant *k*_0_ = 100, the equilibrium bond length *r*_0_ = 0.1, the bending constant *k*_angle_ = 90 and *θ*_0_ = 0.0. The backbone of SCLCP is considered as a bead-spring chain with *k*_0_ = 100 and *r*_0_ = 1.0, where *r*_0_ is the equilibrium bond length between the center of mass of two adjacent beads.

### 2.3 Simulation Settings

To study the LC phase transition of mesogens in different molecules, simulations are in NVT ensemble with periodic boundary condition is used. For mesogens in small molecular LC systems, the simulations are performed with 1000 mesogens. For MCLCP systems, they contain 100 polymer chains and each has 10 mesogens. For SCLCP systems, there are 10 mesogens and 19 beads in 64 polymer chains. These three system have the same volume (*V =* 15*σ*_0_×15*σ*_0_×15*σ*_0_) and volume fraction of particles (ρ*≈0.47). Further, to confirm finite size effect in the simulation systems, we also carried out simulation in the simulation box with double size. The large systems contain 8000 mesogens for small molecular LC and MCLCP with 20 mesogens in each chain, as well as 4960 mesogens and 9672 beads for SCLCP with 20 mesogens and 39 beads in each chain. A standard leap-frog algorithm for anisotropic systems is used to solve the equations of motion, and the dimensionless MD time step dt* = (*mσ*_0_^2^/*ε*_0_)^1/2^*dt* = 0.001. The reduced temperature T* = *k*_B_*T*/*ε*_0_ is controlled using Nose-Hoover thermostat with a relaxation time *τ*_Τ_ = 0.1, where *k*_B_ is the Boltzmann constant. To observe the phase behavior of the model systems, the simulated annealing process is applied to gradually cool down the systems from equilibrated isotropic melts. Systems are equilibrated for at least 1×10^7^ and 2×10^7^ time steps for mesogens in small molecules and polymers, respectively. The mean properties are represented by statistical averages over ten samples evenly taken from the last 3×10^6^ time steps. To test the simulation cost in small molecule LC systems, the number of particles range from 1×10^3^ to 1×10^6^, and accordingly system sizes from 10*σ*_0_×10*σ*_0_×30*σ*_0_ to 100*σ*_0_×100*σ*_0_×300*σ*_0_ to keep the constant number density *ρ* = *N*/*V* = 0.33. All simulations using either GALAMOST or LAMMPS are performed in single-precision.

Simulations were carried out using the GPU-accelerated package GALAMOST [57] and the well known LAMMPS package [69] for simulation cost comparison. A server equipped four NVIDIA Tesla K20C GPUs (each 2496 cores) and two Intel Xeon E5-2687w CPUs with 128G RAM is used. Each simulation trajectory utilizes either one GPU or CPU.

### 2.4 Characteristic Parameters

The global orientation of mesogens in the simulation system are viewed from the order parameter *S*, which is computed by^3^

$$S=\frac{1}{2N}\sum_{i=1}^{N}\left(3{\text{cos}}^{2}\gamma_{i}-1\right)$$ (8)

The value of *S* is in the range of 0 to 1, 0 for random distributed and 1 for completely orientated mesogens. The angle γ_*i*_ is between the major axis of the mesogen *i* and the principal axis of all mesogens. The principal axis is determined from the largest positive eigenvalue of the second-rank symmetric tensor *Q* ^3,41^,

$$Q_{\alpha \beta }=\sum_{i=1}^{N}\left(\frac{3}{2}u_{i\alpha }u_{i\beta }-\frac{1}{2}\delta_{\alpha \beta }\right)\;\;\alpha ,\beta \in (x,y,z)$$ (9)

where the unit vector **u**_*i*_ points along the major axis of molecule *i*. The Kronecker delta function *δ*_*αβ*_ is 1 when *α* and *β* are identical, and 0 otherwise.

The spatial location of mesogens are indicated from the radial distribution functions *g*(r) of mesogens, which gives the probability of finding a particle in the distance *r* from another particle ^41^,

$$g(r)=\frac{V}{N^{2}}<\sum_{i=1}^{N}\sum_{j\neq i}^{N}\delta (r-r_{ij})>$$ (10)

The range of *g*(r) is from 0 to large positive number. It equals 1 indicates random distribution, larger value for enrichment and less than 1 for depletion of mesogens at given separation range.

Then the orientational correlation of mesogens at given separation is presented by *g*_2_(*r*), defined as ^42^

$$g_{2}(r)=<3{\left[u_{i}(r_{i})\cdot u_{j}(r+r_{i})\right]}^{2}-1>/2$$ (11)

The value of *g*_2_(*r*) equals to 1 corresponds to that all mesogens are in side-by-side or end-to-end packing and a value close to 0 indicates mesogens are in isotropic phase.

The association of mesogens in LC systems can be viewed by the second virial coefficient *A*_2_, which is calculated using the definition of [70]

$$A_{2}=\frac{1}{N_{ij}}\sum_{i=1}^{N}\sum_{j\neq i}^{N}r_{ij}{}_{}^{2}\{1-exp[-U(i,\;j)/k_{B}T]\}$$ (12)

where *N*_*ij*_ and *U*(*i*, *j*) is the total number of interaction pairs and the interaction energy between two particles *i* and *j*, respectively. A negative *A*_2_ indicates that mesogens prefer close packing, and a positive one corresponds to dispersed state.

### 2.5 Protocol and Implementation in GALAMOST

To eliminate the time-consuming data transfer between host memory and device memory, GALAMOST is designed to implement all MD computations on GPUs, including integration, building neighbor list, and the calculation of non-bonded and bonded interactions, etc. By continuous optimization, GALAMOST can run efficiently on a single GPU. Some advanced optimization techniques are employed, such as the SFCPACK sorting method which rearranges the order of data of particles stored in host and device memories to increase the probability of cache hit at memory reading. In addition, the GPU-accelerated neighbor list building algorithm is used to further improve the computational efficiency.

We employ Compute Unified Device Architecture (CUDA) to harness the computational power of GPUs. All GPU threads execute the same instruction, while process different data. The execution mode is thereby called single-instruction-multiple-data (SIMD). We usually design the GPU-accelerated MD algorithm by the principle that one GPU thread tackles the computational task of one particle. GB interactions as non-bonded interactions are calculated in “one thread one particle” mode. The algorithm is to use a neighbor list that lists all interacting GB particles for each particle, built beforehand. Because of the independence of parallel GPU threads, a pair of interacting particles is inevitably separated in neighbor list. The GB forces and torques for each particle are calculated and summed by a corresponding thread on the GPU via searching the particles within cutoff in neighbor list process.

## 3. Results and Discussion

### 3.1 Compare Simulation Cost and Accuracy of MD Simulation on Small Molecular Liquid Crystals

**Fig 3:**
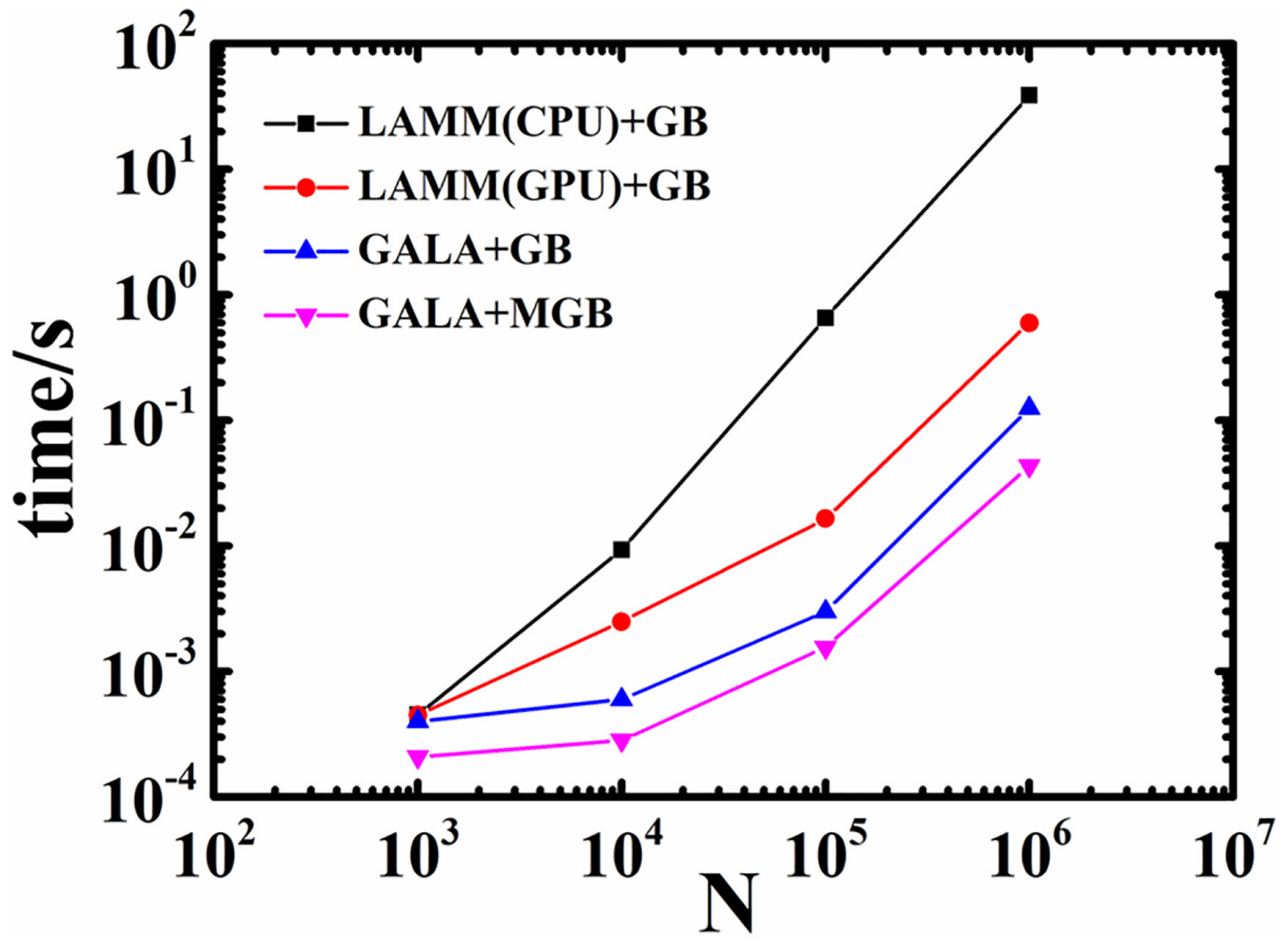
The average simulation cost per step of GALAMOST (GALA) and LAMMPS (LAMM) with the GB (+GB) or the MGB (+MGB) interaction as a function of the number of particles in simulation systems.

Simulation cost is compared between LAMMPS and GALAMOST in the original and modified GB potential forms, with systems spanning from 10^3^ to 10^6^ particles. The average simulation time over 10 parallel runs against the number of mesogens is presented in Fig 3. For small system (*N*<10^3^), the GPU-acceleration is not prominent compared with conventional CPU-based algorithm. When the number of particles reaches 10^6^, the cost of GPU MD simulation using GALAMOST were about 4 times and more than 200 times less compared the GPU and CPU MD simulation in LAMMPS, respectively. Compared with the original GB potential, the MGB potential which takes a simplified functional form affords about 30% to 50% in the reduction of simulation cost.

**Fig 4:**
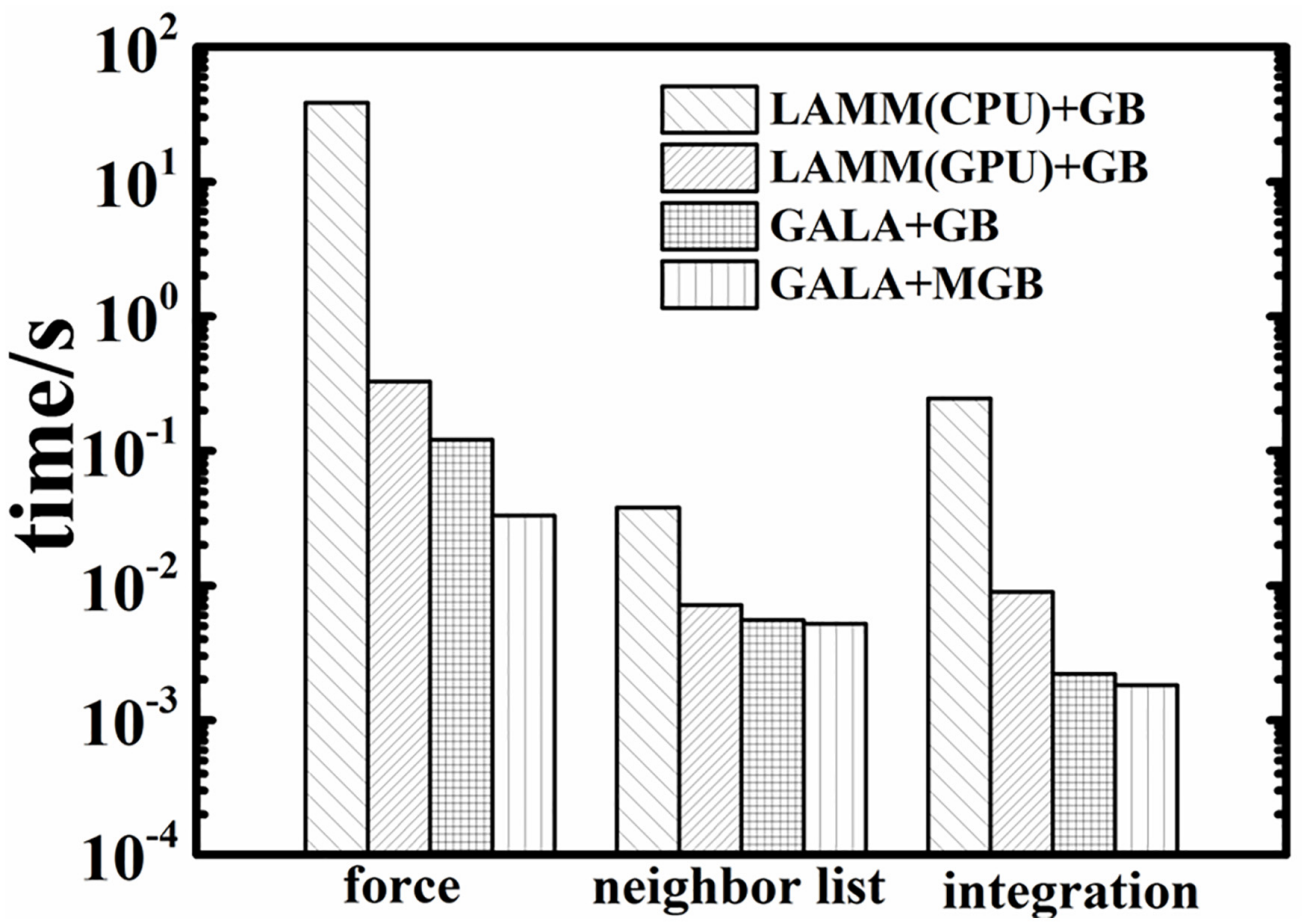
The average simulation cost per step of GALAMOST and LAMMPS for pair force, neighbor list and integration, the three major time consuming functions in MD simulation.

We also decoupled the three major time-consuming processes and their comparisons for a simulation system with 10^6^ particles are shown in Fig 4. The GPU-accelerated simulation show advantages in all the three functions of force, neighbor list and integration processes. Because of the high complexity of GB potential, force calculation turns to be the major time consuming process. The success of GPU acceleration may benefit from two important aspects: (1) the computational power of a single GPU, such as Tesla K20C with a theoretical peak 3.95 Teraflops of single precision computation throughput, is usually more than a hundred times faster than that of a CPU core, such as Xeon E5-2687w with 21.56 Gigaflops per core; (2) GALAMOST is highly optimized such as the memory sorting technique which can significantly reduce the time for nonbonded force calculation, and the elimination of inter-memory communication[57].

**Fig 5:**
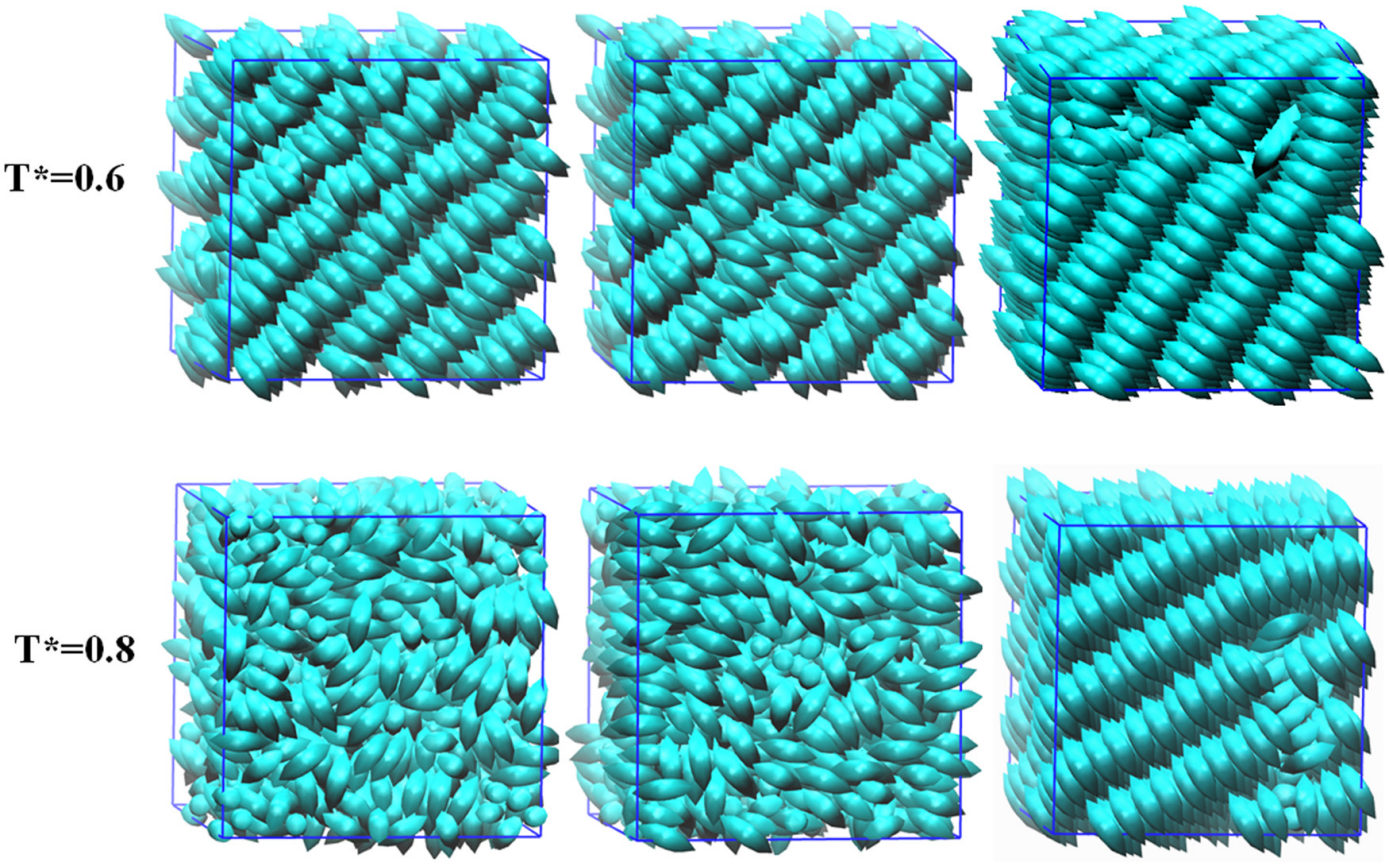
Equilibrium configurations of typical phases with the GB interaction at various temperatures T* = 0.6 and T* = 0.8 obtained by GALAMOST with GB (left), LAMMPS with GB (middle), and GALAMOST with MGB (right).

The snapshots of simulation configurations under equilibrium from low to high temperatures, associated with the phase transition of small molecular LC are presented in Fig 5. At low temperature (T* = 0.6), mesogens are condensed in a smectic-B phase. The global orientation of mesogens is conservative for the simulation guided by GB potential, independent on either the CPU-approach used in LAMMPS or the GPU-acceleration approach implemented in GALAMOST. At high temperature (T* = 0.8), there is no global orientation, exhibiting typical isotropic phase. The configurations based on GB interaction produced by the two simulation packages are non-distinguishable, while the simulation guided by MGB which has degenerate states of side-by-side and cross interactions did not show the orientational transition at this temperature window.

**Fig 6:**
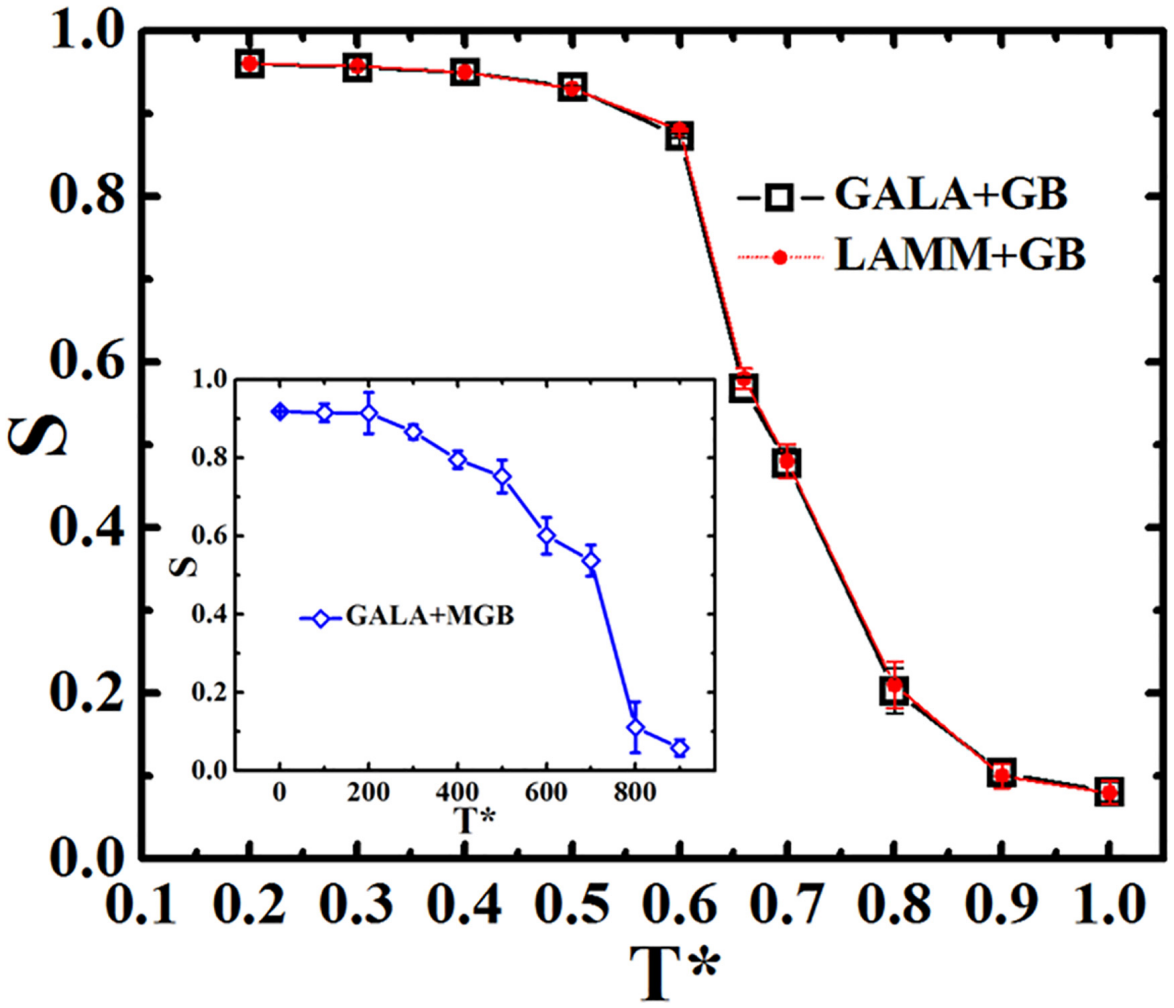
Orientational order parameter *S* of mesogens in small molecules as a function of temperature and simulation approaches.

Further comparison of simulation accuracy through the global order parameter *S* is shown in Fig 6. The temperature dependence of the order parameter from the configurations generated by GB potential with GALAMOST and LAMMPS packages are almost identical. At low temperature, *S* levels off at 0.96, indicating that measogens are highly orientated. At a critical temperature at T* = 0.66, there is a smectic-isotropic phase transition. At high temperature (T*>0.9), the value of *S* is close to 0, meaning that globular orientation of mesogens vanishes. However, the phase transition guided by MGB spanned in a much broader temperature window. It suggests that the MGB potential is not suitable for LC phase transition study although it has merits in simulation cost saving.

**Fig 7:**
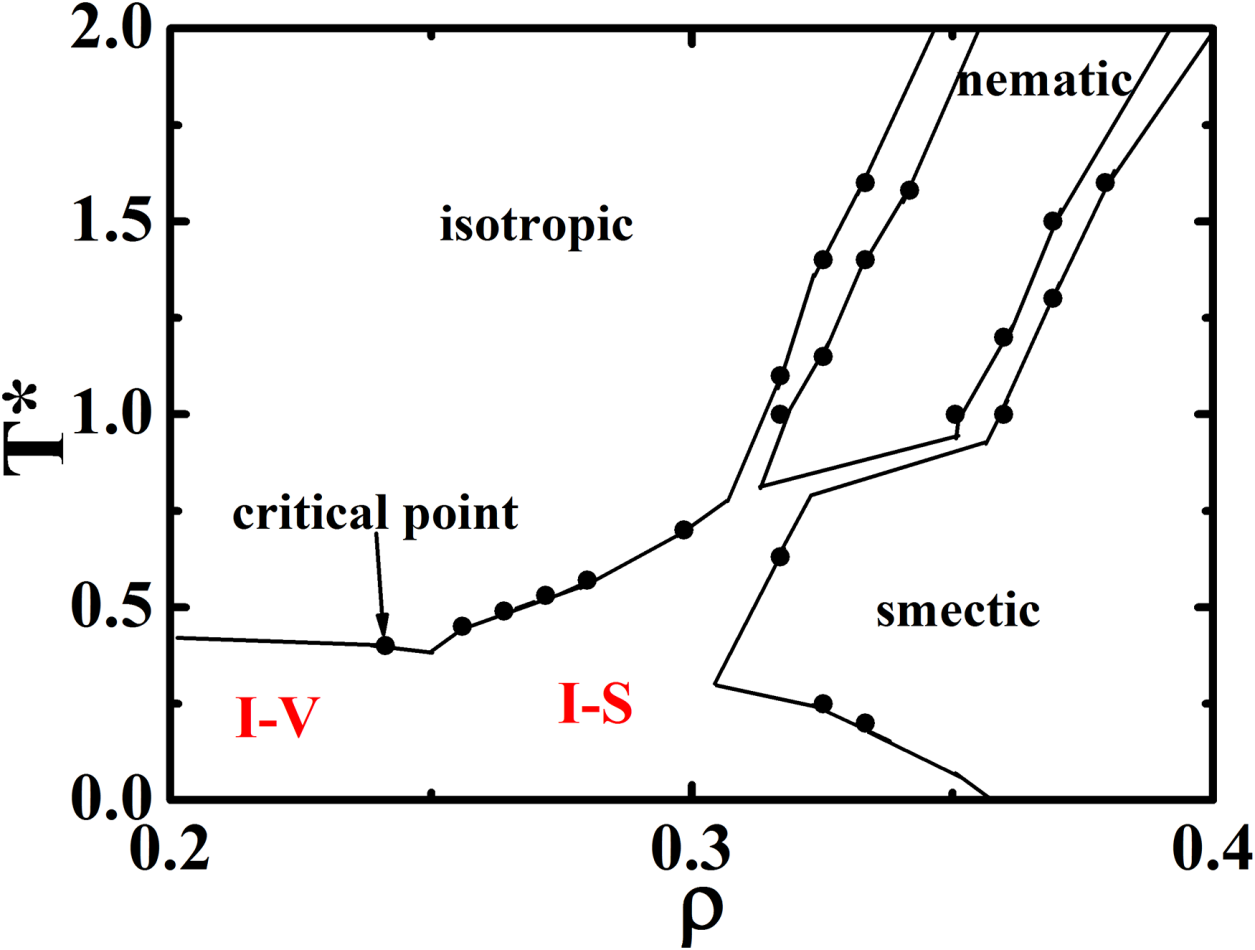
The phase diagram of mesogens in small molecular LC obtained by GPU-accelerated simulation equipped with coarse grained GB potential. Solid circles mark our simulation results and lines are plotted for guide only. The X-axis is converted to number density for the comparison with de Miguel’s report.

To further confirm the accuracy of GALAMOST and GB potential in the simulation of LC phase transitions, we reproduced the phase diagram (Fig 7) using the identical simulation systems reported by de Miguel *et al*. [36]. The data points to identify the phase boundary are determined from configurations generated in simulated annealing processes. Either the global order parameter *S* sharply increases or the *g*(*r*) profile has new peaks (it is also combined with direct visualization of configurations) is regarded as a phase transition point. Using this criterion, the GPU-accelerated MD simulation equipped with GB potential can reliably and accurately repeat the phase diagram. Therefore, in the following section, the LC phase transitions for mesogens in different molecules are only simulated by the GALAMOST package with GB potential.

### 3.2 Liquid Crystal Phase Transition of Mesogens in Different Molecules

**Fig 8:**
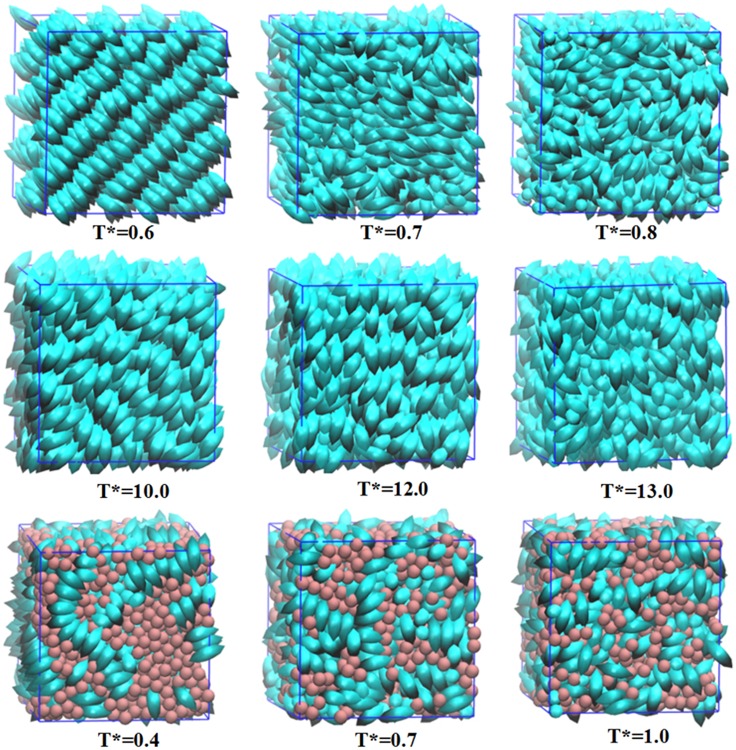
Snapshots of typical phases for mesogens in small molecules (up), MCLCP (middle) and SCLCP (bottom) with temperature increasing from left to right.

Typical snapshots of simulation configurations for small molecules, MCLCP and SCLCP at ordered, intermediate and disordered states with temperature increasing are shown in Fig 8. At high temperature, the location and the orientational orders disappeared, all systems exhibit typically isotropic phases. At low temperature, mesogens in small molecules are packed in smectic phases with layered and ordered structures, while nematic phases appear in both SCLCP and MCLCP systems. Although small molecules and MCLCP have the same number of mesogens, the competition between the orientational ordering of mesogens and the entropy maximization of the backbone in MCLCP leads to higher transition temperature than that in small molecular systems. Compared with the one-step transition from liquid to ordered LC phase for mesogens in small molecules and MCLCP, SCLCP undergo two-step for this transition as temperature decreases. When temperature decreases from 1.0 to 0.7, mesogens in SCLCP gradually assembled into enriched domains. In this process, nematic nuclei are formed and rapidly grow in size (see S1 Fig). Further decreasing to low temperature (T* = 0.4), SCLCP forms multi-domain nematic phase where mesogens in each domain has a favored orientation. It is due to that the backbones and spacers hinder the rotational orientation of the mesogenes. The phase transition for SCLCP in this simulation is consistent with the results in birefringence and optical transmission experiments [46, 47]. Boamfa *et al*. studied various SCLCPs and found that during the first-order isotropic-nematic phase transition, the favored orientation for each domain is determined by the ordering fluctuation which originates from the polymer backbone coupling effect.

**Fig 9:**
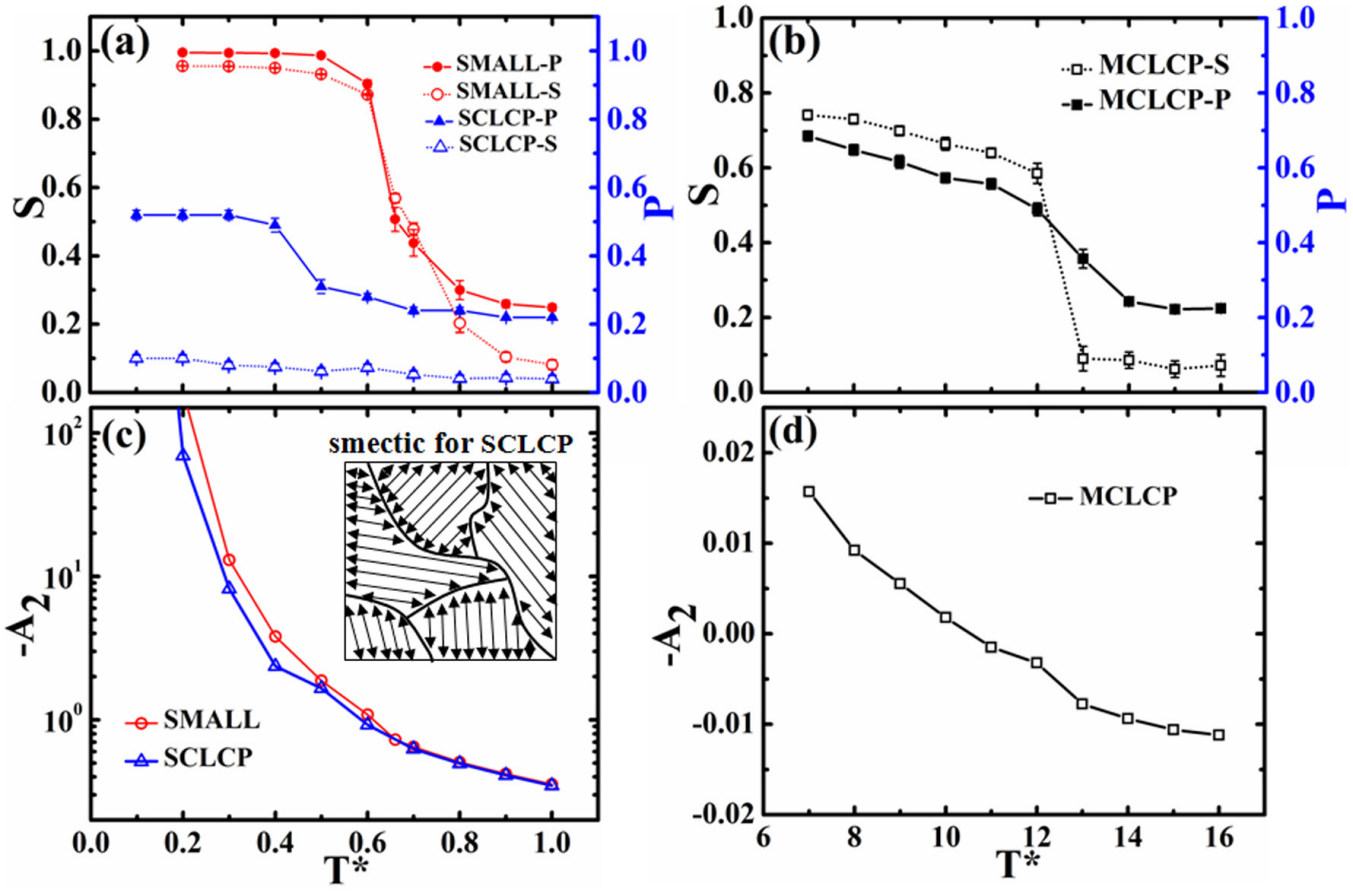
The orientational order parameter *S*, the probability of the local orientation *P* (a, b), and the second virial coefficient *A*_2_ (c, d) as a function of temperature for mesogens in small molecular, SCLCP and MCLCP systems. Insert in (c) illustrated the multi-domain nematic phase for SCLCP system.

The orientational order parameter *S* for mesogens in small molecules, SCLCP and MCLCP systems are shown in Fig 9(a) and 9(b). The temperature dependence of *S* for both small molecules and MCLCP follow a Sigmoidal curve [71] associated with the LC phase transition. Compared to the sharp transition of small molecular LC, MCLCP spans a broader temperature window and the critical transition temperature is at a higher temperature T* = 12.0. The LC transition of MCLCP may experience an coexistence of anisotropic and isotropic phases, as experimentally revealed by Shilov *et al*. [72] The higher critical temperature for MCLCP results from the restricted translational and rotational motions of the mesogens by the rigid backbone, and the additional energy penalty derived from the orientation of polymer backbones[73]. SCLCP shows multi-domain morphology with local orientation as indicated from the global order parameter *S* is nearly 0. Correspondingly, in experiment, the birefringence of this multi-domain morphology is very small due to the absence of a common director orientation. Furthermore, in order to show the degree of local orientation, we propose the probability *P* to count the preference of local ordering composed by neighboring mesogens. The probability *P* is defined as proportion of the angle between their major axes satisfies **|**cos(**u**_*i*_·**u**_*j*_)**|**>0.8 outcomes to the total number when two mesogens with separation distance less than 3.4*σ*_0_. For small molecules and MCLCP systems, the temperature dependence of *P* matches *S* well. However, as SCLCP only has local orientation, *S* does not reveal the corresponding ordering associated with the LC transition, while *P* clearly shows such transition.

We also calculated the second virial coefficient *A*_2_ to view the association preference of mesogens, as shown in Fig 9(c) and 9(d). Increasing temperature resulting in the decrease of -*A*_2_, which can be seen in all three systems. It may be attributed to the decrease of attractive interaction and the reduction of closely packed neighboring molecules according to the definition of *A*_2_. Further, due to the extended conformation of rigid main-chain and higher temperature zone, *A*_2_ of MCLCP is much smaller than those in small molecular and SCLCP systems. At low temperatures, strong interactions among mesogens can assemble almost all molecules together, and mesogens in all three systems show an ordered structure and a close-packed arrangement. At high temperatures, the asymptotic value of *A*_2_ is close to 0 in small molecular and SCLCP systems, suggesting the ordered packing of mesogens is not favored. In other words, mesogens are in isotropic state. However, the *A*_2_ in MCLCP systems becomes positive. This is due to that the location of mesogens in MCLCP system is disordered, and mesogens prefer to orient along the rigid main chain and repel each other. It has also been reported by Withers *et al*.[74] that the second virial coefficient gradually increases to positive with temperature increasing for rigid polymers. Increasing temperature weakens the attractive interaction associated with the packing of orientated mesogens, and the rigidity of the MCLCP backbone leads to slightly repulsion among mesogens.

**Fig 10:**
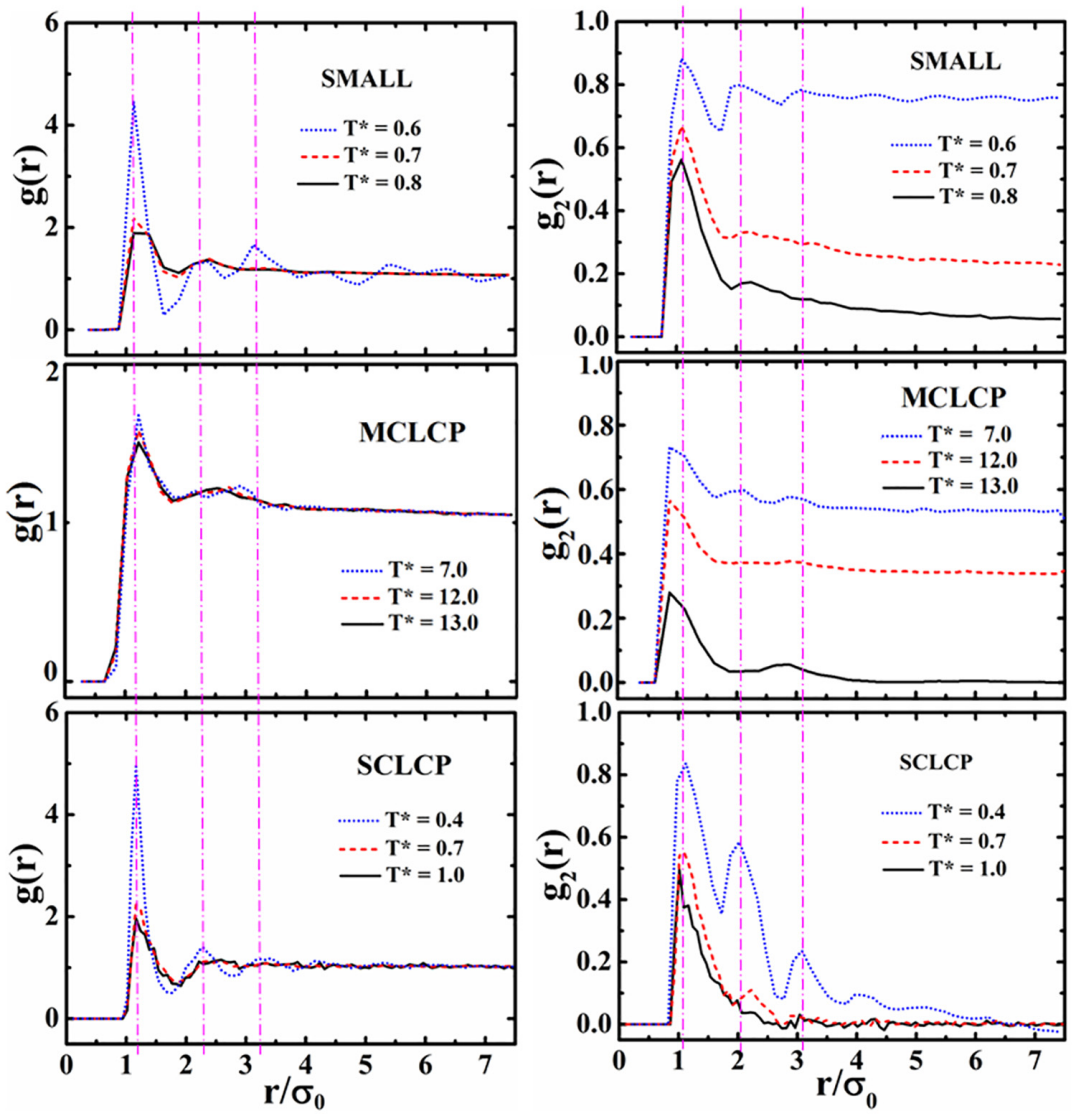
The radial distribution function *g*(*r*) and the orientational correlation functions *g*_2_(*r*) at various temperatures for small molecular, MCLCP and SCLCP systems. The three vertical dash lines label the location of the side-by-side and cross, the 2^nd^ nearest side-by-side and T-shape, and the end-to-end packing of mesogens from left to right.

To further clarify the distribution and the orientation of mesogens, the radial distribution function *g*(*r*) and the orientational correlation function *g*_2_(*r*) are presented in Fig 10. All systems show an obvious first correlation peak at 1.12σ_0_ which corresponds to side-by-side and cross packing of mesogens, and the former is the majority as shown from the positive peak values of *g*_2_(*r*). The second and third peaks are two and three times the separation distance of the first peak, respectively. It indicates that the packing of mesogens achieve long-range ordering. Further decreasing temperature makes the correlation peaks more prominent, in good agreement with that lower temperature leads to higher ordering of thermotropic LC. The value of *g*_2_(*r*) at long distances tends to *S*^2^, which is consistent with the results of simulation and theory ^3^. Different from mesogens in small molecules, there is no sign of long-range positional order for MCLCP at low temperature (T* = 7.0). The curve of *g*_2_(*r*) shows the nearest-neighbor peak (*r/*σ_0_≈1.12) and level-off at *S*^2^ when mesogens are separated far enough. It indicated that the system appears to form a nematic, not a smectic phase. In the isotropic phase (T* = 13.0), *g*_2_(*r*) decays to zero at large separations and a small peak (*r/*σ_0_≈3) reflects the strong intramolecular correlations. Compared with uniform orientation in nematic phase, there is no sign of long-range orientational order. For SCLCP, at low temperature (T* = 0.4), the curve of *g*(*r*) show a peak at *r/*σ_0_≈1.12 for the closely packed neighboring mesogens. There is no obvious peak at 3*σ*_0_ because the end-to-end packing of mesogens is inhibited by the backbone in SCLCP. Compared both *g*(*r*) and *g*_2_(*r*) for mesogens in polymers with those in small molecules, the connection of polymer chain can efficiently screen the long range correlation of orientated mesogens. Finally, in LC phase, the side-by-side and the end-to-end are favorable, and the cross and the T-shape have low occurrences.

Additionally, the finite size effect on the simulation of LC phase transition is also studied. The results from the simulation of large systems are presented in S2 and S3 Figs. Compared with the results shown above, it can be seen that enlarging simulation box only slightly changes the absolute values. The order parameter *S*, the probability of the local orientation *P*, the secondary Virial coefficients *A*_2_ and the correlation functions all show similar trend. These results suggest that the simulation system in this work is large enough to present reliable description for LC phase transition for mesogens in different molecules. Meanwhile, we also found that longer polymer chain causes MCLCP to form ordered structure at lower temperature, while SCLCP has phase transition at slightly higher temperature.

**Fig 11:**
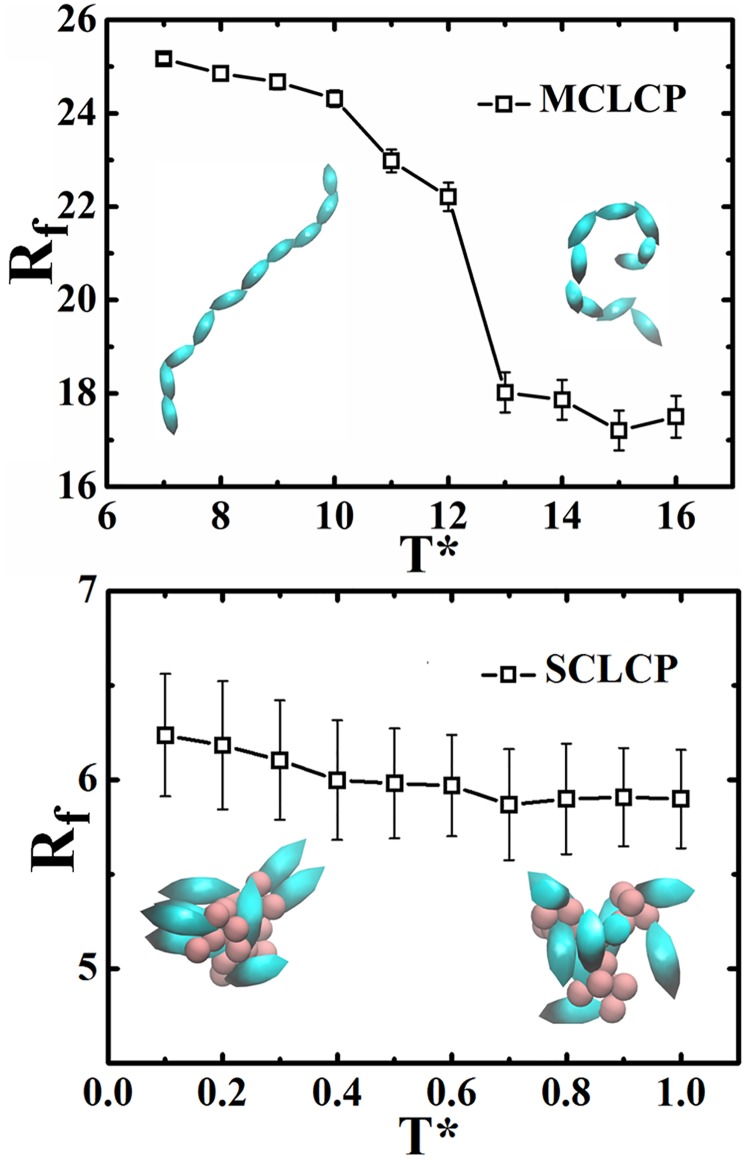
The average end-to-end distance (*R*_*f*_) of backbone for MCLCP and SCLCP.

In order to give a deep understanding in phase transition in LCP systems, the change of conformations for individual polymer chains along with temperature, characterized by the average end-to-end distance (*R*_*f*_) for polymer backbones in MCLCP and SCLCP systems, is presented in Fig 11. The *R*_*f*_ of MCLCP sharply decreases from T* = 12.0 to T* = 13.0, suggesting that chain collapse is coupled with the isotropic-nematic phase transition. The chain collapse results in less orientation among intra-chain mesogens, as revealed from the decrease in *g*_2_(*r*). In contrast, on cooling, the shape of the backbone of SCLCP is always a coil with *R*_*f*_ only slightly increasing, which is associated with the multi-domain isotropic-nematic transition. Such increasing originates from that polymer chains spanned multiple orientated domains. In additional, assuming that polymers are Gaussian, the ideal *R*_*f*_ values are 9.5σ_0_ and 4.4σ_0_ for MCLCP and SCLCP, respectively. The actual *R*_*f*_ values under all conditions are significantly larger, indicating that the presence of mesogens extends polymer backbones.

## 4. Conclusion

We developed GPU-accelerated Molecular Dynamics simulations combined with coarse-grained Gay-Berne potential to investigate the temperature induced liquid crystal phase transition of mesogens in small molecules, main-chain liquid crystal polymers and side-chain liquid crystal polymers. On cooling, mesogens in small molecular liquid crystals exhibit the isotropic-smectic transition, main-chain liquid crystal polymers assemble into nematic phases, and mesogens in side-chain liquid crystal polymers undergo a two-step process including nucleation of nematic islands and formation of multi-domain nematic texture. Mesogens in small molecules and side-chain liquid crystal polymers share lower phase transition temperature, and main-chain liquid crystal polymers need much higher temperature to overcome the energy barrier from the orientation of rigid polymer backbones.

For systems containing up to one million particles, the GPU-accelerated simulation in GALAMOST is about 4 times less computational cost than the GPU simulation, and at least 200 times faster than the conventional CPU simulation in LAMMPS. The acceleration is mainly contributed from the efficient handling of the high computation complexity of Gay-Berne potential, which is the major time-consuming process. The modified form of Gay-Berne potential is confirmed not suitable in study of liquid crystal phase transition though it has merit to reduce the computational complexity. The GPU-accelerated MD simulation with GB potential implemented in GALAMOST package is efficient and accurate to study the complex phase transition in liquid crystal systems. It provides a useful tool to simulate anisotropic particles and interactions for larger system size over longer time.

## Supporting Information

S1 Fig Snapshots of typical phases for SCLCP. (DOC)

S2 Fig The orientational order parameter, the probability of the local orientation, and the second virial coefficient. (DOC)

S3 Fig The radial distribution function and the orientational correlation function. (DOC)

## References

[1] Allen MP, Brown JT, Warren MA. Computer simulation of liquid crystals. J Phys-Condens Mat. 1996;8:9433–7.

[2] Juszynska-Galazka E, Galazka M, Massalska-Arodz M, Bak A, Chledowska K, Tomczyk W. Phase Behavior and Dynamics of the Liquid Crystal 4 '-butyl-4-(2-methylbutoxy)azoxybenzene (4ABO5*). J Phys Chem B. 2014;118:14982–9. 25429851 10.1021/jp510584w

[3] Lyulin AV, Al-Barwani MS, Allen MP, Wilson MR, Neelov I, Allsopp NK. Molecular dynamics simulation of main chain liquid crystalline polymers. Macromolecules. 1998;31:4626–34.

[4] Sasaki H, Takanishi Y, Yamamoto J, Yoshizawa A. Supermolecular Bent Configuration Composed of Achiral Flexible Liquid Crystal Trimers Exhibiting Chiral Domains with Opposite Handedness. J Phys Chem B. 2015;119:4531–8. 10.1021/jp512710r 25724005

[5] Zhou ZX, Wang JH, Yu JC, Shen YF, Li Y, Liu AR, et al Dissolution and Liquid Crystals Phase of 2D Polymeric Carbon Nitride. J Am Chem Soc. 2015;137:2179–82. 10.1021/ja512179x 25634547

[6] Ware TH, McConney ME, Wie JJ, Tondiglia VP, White TJ. Voxelated liquid crystal elastomers. Science. 2015;347:982–4. 10.1126/science.1261019 25722408

[7] Vita F, Sparnacci K, Panzarasa G, Placentino IF, Marino S, Scaramuzza N, et al Evidence of Cybotactic Order in the Nematic Phase of a Main-Chain Liquid Crystal Polymer with Bent-Core Repeat Unit. Acs Macro Lett. 2014;3:91–5. 10.1021/mz400518x 35651116

[8] Koga M, Sato K, Kang SM, Sakajiri K, Watanabe J, Tokita M. Influence of Smectic Liquid Crystallinity on Lamellar Microdomain Structure in a Main-Chain Liquid Crystal Block Copolymer Fiber. Macromol Chem Phys. 2013;214:2295–300.

[9] Martinez-Felipe A, Imrie CT, Ribes-Greus A. Study of Structure Formation in Side-Chain Liquid Crystal Copolymers by Variable Temperature Fourier Transform Infrared Spectroscopy. Ind Eng Chem Res. 2013;52:8714–21.

[10] Chen XF, Shen ZH, Wan XH, Fan XH, Chen EQ, Ma YG, et al Mesogen-jacketed liquid crystalline polymers. Chem Soc Rev. 2010;39:3072–101. 10.1039/b814540g 20559597

[11] Yang G, Tang P, Yang YL. Uniaxial-Biaxial Nematic Phase Transition in Combined Main-Chain/Side-Chain Liquid Crystal Polymers Using Self-Consistent Field Theory. Macromolecules. 2012;45:3590–603.

[12] Care CM, Cleaver DJ. Computer simulation of liquid crystals. Rep Prog Phys. 2005;68:2665–700.

[13] Wilson MR. Molecular simulation of liquid crystals: Progress towards a better understanding of bulk structure and the prediction of material properties. Chem Soc Rev. 2007;36:1881–8. 17982515 10.1039/b612799c

[14] Liu AJ, Grest GS, Marchetti MC, Grason GM, Robbins MO, Fredrickson GH, et al Opportunities in theoretical and computational polymeric materials and soft matter. Soft Matter. 2015;11:2326–32. 10.1039/c4sm02344g 25711605

[15] Dicke HR, Lenz RW. Liquid-Crystal Polymers .20. Poly(1,4-Cycloalkylhydroquinone Terephthalates) with Flexible Hexamethylene Spacers in the Main Chain. Angew Makromol Chem. 1985;131:95–105.

[16] Gay JG, Berne BJ. Modification of the Overlap Potential to Mimic a Linear Site-Site Potential. J Chem Phys. 1981;74:3316–9.

[17] Shen HJ, Li Y, Ren PY, Zhang DL, Li GH. Anisotropic Coarse-Grained Model for Proteins Based On Gay-Berne and Electric Multipole Potentials. J Chem Theory Comput. 2014;10:731–50. 24659927 10.1021/ct400974z PMC3958967

[18] Peroukidis SD, Lichtner K, Klapp SHL. Tunable structures of mixtures of magnetic particles in liquid-crystalline matrices. Soft Matter. 2015;11:5999–6008. 10.1039/c5sm00903k 26041553

[19] van Meel JA, Arnold A, Frenkel D, Zwart SFP, Belleman RG. Harvesting graphics power for MD simulations. Mol Simulat. 2008;34:259–66.

[20] Anderson JA, Lorenz CD, Travesset A. General purpose molecular dynamics simulations fully implemented on graphics processing units. J Comput Phys. 2008;227:5342–59.

[21] Stone JE, Hardy DJ, Ufimtsev IS, Schulten K. GPU-accelerated molecular modeling coming of age. J Mol Graph Model. 2010;29:116–25. 10.1016/j.jmgm.2010.06.010 20675161 PMC2934899

[22] Rovigatti L, Gnan N, Parola A, Zaccarelli E. How soft repulsion enhances the depletion mechanism. Soft Matter. 2015;11:692–700. 10.1039/c4sm02218a 25428843

[23] Nielsen SO, Lopez CF, Srinivas G, Klein ML. Coarse grain models and the computer simulation of soft materials. J Phys-Condens Mat. 2004;16:R481–R512.

[24] Gemunden P, Daoulas KC. Fluctuation spectra in polymer nematics and Frank elastic constants: a coarse-grained modelling study. Soft Matter. 2015;11:532–44. 10.1039/c4sm02075h 25418080

[25] Cuetos A, Martinez-Haya B, Rull LF, Lago S. Monte Carlo study of liquid crystal phases of hard and soft spherocylinders. J Chem Phys. 2002;117:2934–46.

[26] Dewar A, Camp PJ. Computer simulations of bent-core liquid crystals. Phys Rev E. 2004;70:011704. 10.1103/PhysRevE.70.011704 15324069

[27] Workineh ZG, Vanakaras AG. Surface-Induced Ordering on Model Liquid Crystalline Dendrimers. Polymers-Basel. 2014;6:2082–99.

[28] Heinemann T, Palczynski K, Dzubiella J, Klapp SHL. Angle-resolved effective potentials for disk-shaped molecules. J Chem Phys. 2014;141:214110 10.1063/1.4902824 25481132

[29] Busselez R, Cerclier CV, Ndao M, Ghoufi A, Lefort R, Morineau D. Discotic columnar liquid crystal studied in the bulk and nanoconfined states by molecular dynamics simulation. J Chem Phys. 2014;141:134902 10.1063/1.4896052 25296832

[30] Qi WK, Xu Y, Yung KL, Chen Y. A modified Gay-Berne model for liquid crystal molecular dynamics simulation. Polymer. 2012;53:634–9.

[31] Bose TK, Saha J. Monte Carlo Simulations of Spontaneous Ferroelectric Order in Discotic Liquid Crystals. Phys Rev Lett. 2013;110:265701 23848900 10.1103/PhysRevLett.110.265701

[32] Ebrahimi D, Whittle AJ, Pellenq RJM. Mesoscale properties of clay aggregates from potential of mean force representation of interactions between nanoplatelets. J Chem Phys. 2014;140:154309.

[33] Satoh K. Thermodynamic scaling of dynamic properties of liquid crystals: Verifying the scaling parameters using a molecular model. J Chem Phys. 2013;139:084901 10.1063/1.4818418 24007031

[34] Ilnytskyi JM, Neher D. Structure and internal dynamics of a side chain liquid crystalline polymer in various phases by molecular dynamics simulations: A step towards coarse graining. J Chem Phys. 2007;126:174905 17492884 10.1063/1.2712438

[35] de Miguel E, Vega C. The global phase diagram of the Gay-Berne model. J Chem Phys. 2002;117:6313–22.

[36] Demiguel E, Rull LF, Chalam MK, Gubbins KE. Liquid-Crystal Phase-Diagram of the Gay-Berne Fluid. Mol Phys. 1991;74:405–24.

[37] Luckhurst GR, Simmonds PSJ. Computer-Simulation Studies of Anisotropic Systems .21. Parametrization of the Gay-Berne Potential for Model Mesogens. Mol Phys. 1993;80:233–52.

[38] Shibaev VP. Liquid-Crystalline Polymer Systems: From the Past to the Present. Polym Sci Ser a+. 2014;56:727–62.

[39] Ilnytskyi J, Wilson MR. A domain decomposition molecular dynamics program for the simulation of flexible molecules with an arbitrary topology of Lennard-Jones and/or Gay-Berne sites. Comput Phys Commun. 2001;134:23–32.

[40] Ilnytskyi JM, Wilson MR. A domain decomposition molecular dynamics program for the simulation of flexible molecules of spherically-symmetrical and nonspherical sites. II. Extension to NVT and NPT ensembles. Comput Phys Commun. 2002;148:43–58.

[41] McBride C, Wilson MR. Molecular dynamics simulations of a flexible liquid crystal. Mol Phys. 1999;97:511–22.

[42] Wilson MR. Molecular dynamics simulations of flexible liquid crystal molecules using a Gay-Berne/Lennard-Jones model. J Chem Phys. 1997;107:8654–63.

[43] Stimson LM, Wilson MR. Molecular dynamics simulations of side chain liquid crystal polymer molecules in isotropic and liquid-crystalline melts. J Chem Phys. 2005;123:034908. 10.1063/1.1948376 16080764

[44] Pujolle-Robic C, Noirez L. Observation of shear-induced nematic-isotropic transition in side-chain liquid crystal polymers. Nature. 2001;409:167–71. 11196635 10.1038/35051537

[45] Gopinadhan M, Majewski PW, Choo Y, Osuji CO. Order-Disorder Transition and Alignment Dynamics of a Block Copolymer Under High Magnetic Fields by In Situ X-Ray Scattering. Phys Rev Lett. 2013;110:078301 25166413 10.1103/PhysRevLett.110.078301

[46] Boamfa MI, Viertler K, Wewerka A, Stelzer F, Christianen PCM, Maan JC. Mesogene-polymer backbone coupling in side-chain polymer liquid crystals, studied by high magnetic-field-induced alignment. Phys Rev Lett. 2003;90:025501 12570554 10.1103/PhysRevLett.90.025501

[47] Boamfa MI, Viertler K, Wewerka A, Stelzer F, Christianen PCM, Maan JC. Magnetic-field-induced changes of the isotropic-nematic phase transition in side-chain polymer liquid crystals. Phys Rev E. 2003;67:050701. 10.1103/PhysRevE.67.050701 12786124

[48] Vanzo D, Ricci M, Berardi R, Zannoni C. Shape, chirality and internal order of freely suspended nematic nanodroplets. Soft Matter. 2012;8:11790–800.

[49] Skacej G, Zannoni C. Main-chain swollen liquid crystal elastomers: a molecular simulation study. Soft Matter. 2011;7:9983–91.

[50] Persson RAX. Note: Modification of the Gay-Berne potential for improved accuracy and speed. J Chem Phys. 2012;136:226101 10.1063/1.4729745 22713074

[51] Friedrichs MS, Eastman P, Vaidyanathan V, Houston M, Legrand S, Beberg AL, et al Accelerating Molecular Dynamic Simulation on Graphics Processing Units. J Comput Chem. 2009;30:864–72. 10.1002/jcc.21209 19191337 PMC2724265

[52] Yang KD, Bai ZQ, Su JY, Guo HX. Efficient and Large-Scale Dissipative Particle Dynamics Simulations on GPU. Soft Mater. 2014;12:185–96.

[53] Sunarso A, Tsuji T, Chono S. GPU-accelerated molecular dynamics simulation for study of liquid crystalline flows. J Comput Phys. 2010;229:5486–97.

[54] Nguyen TD, Carrillo JMY, Matheson MA, Brown WM. Rupture mechanism of liquid crystal thin films realized by large-scale molecular simulations. Nanoscale. 2014;6:3083–96. 10.1039/c3nr05413f 24264516

[55] Plimpton S. Fast Parallel Algorithms for Short-Range Molecular-Dynamics. J Comput Phys. 1995;117:1–19.

[56] Nguyen TD, Phillips CL, Anderson JA, Glotzer SC. Rigid body constraints realized in massively-parallel molecular dynamics on graphics processing units. Comput Phys Commun. 2011;182:2307–13.

[57] Zhu YL, Liu H, Li ZW, Qian HJ, Milano G, Lu ZY. GALAMOST: GPU-accelerated large-scale molecular simulation toolkit. J Comput Chem. 2013;34:2197–211. 24137668 10.1002/jcc.23365

[58] Li ZW, Lu ZY, Zhu YL, Sun ZY, An LJ. A simulation model for soft triblock Janus particles and their ordered packing. RSC Adv. 2013;3:813–22.

[59] Karimi-Varzaneh HA, Qian HJ, Chen X, Carbone P, Müller-Plathe F. IBIsCO: A molecular dynamics simulation package for coarse-grained simulation. J Comput Chem. 2011;32:1475–87. 10.1002/jcc.21717 21425295

[60] Milano G, Kawakatsu T. Hybrid particle-field molecular dynamics simulations for dense polymer systems. J Chem Phys. 2009;130:214106–8. 10.1063/1.3142103 19508055

[61] Liu H, Zhu YL, Zhang J, Lu ZY, Sun ZY. Influence of Grafting Surface Curvature on Chain Polydispersity and Molecular Weight in Concave Surface-Initiated Polymerization. ACS Macro Letters. 2012;1:1249–53. 10.1021/mz3003374 35607149

[62] Liu Y, Li Y, He J, Duelge KJ, Lu Z, Nie Z. Entropy-Driven Pattern Formation of Hybrid Vesicular Assemblies Made from Molecular and Nanoparticle Amphiphiles. J Am Chem Soc. 2014;136:2602–10. 10.1021/ja412172f 24447129

[63] Wu Z, Li Y, Liu J, Lu Z, Zhang H, Yang B. Colloidal Self-Assembly of Catalytic Copper Nanoclusters into Ultrathin Ribbons. Angew Chem Int Ed. 2014;126:12392–6. 10.1002/anie.201407390 25224070

[64] Xue YH, Zhu YL, Quan W, Qu FH, Han C, Fan JT, et al Polymer-grafted nanoparticles prepared by surface-initiated polymerization: the characterization of polymer chain conformation, grafting density and polydispersity correlated to the grafting surface curvature. Physical Chemistry Chemical Physics. 2013;15:15356–64. 10.1039/c3cp51960k 23928871

[65] Xie SJ, Qian HJ, Lu ZY. Polymer brushes: A controllable system with adjustable glass transition temperature of fragile glass formers. J Chem Phys. 2014;140:044903. 25669577 10.1063/1.4862234

[66] Xie SJ, Qian HJ, Lu ZY. Hard and soft confinement effects on the glass transition of polymers confined to nanopores. Polymer. 2015;142:074902. 10.1063/1.4908047 25702026

[67] Berardi R, Micheletti D, Muccioli L, Ricci M, Zannoni C. A computer simulation study of the influence of a liquid crystal medium on polymerization. J Chem Phys. 2004;121:9123–30. 15527380 10.1063/1.1790453

[68] Allen MP, Tildesley DJ. Computer Simulation of Liquids: Oxford Science Publications; 1989.

[69] Brown WM, Petersen MK, Plimpton SJ, Grest GS. Liquid crystal nanodroplets in solution. J Chem Phys. 2009;130:044901 10.1063/1.3058435 19191407

[70] Li YQ, Huang QR. Influence of Protein Self-Association on Complex Coacervation with Polysaccharide: A Monte Carlo Study. J Phys Chem B. 2013;117:2615–24. 10.1021/jp309135m 23414391

[71] Li YQ, Sun ZY, Shi TF, An LJ. Conformation studies on sol-gel transition in triblock copolymer solutions. J Chem Phys. 2004;121:1133–40. 15260650 10.1063/1.1758938

[72] Shilov S, Birshtein T, Volchek B. Conformational Structure of Liquid-Crystalline Polyesters with Mesogens and Spacers in the Main Chain. Makromol Chem-Theor. 1993;2:21–36.

[73] Blumstein A, Vilasagar S, Ponrathnam S, Clough SB, Blumstein RB, Maret G. Nematic and Cholesteric Thermotropic Polyesters with Azoxybenzene Mesogenic Units and Flexible Spacers in the Main Chain. J Polym Sci Pol Phys. 1982;20:877–92.

[74] Withers IM. Effects of longitudinal quadrupoles on the phase behavior of a Gay-Berne fluid. J Chem Phys. 2003;119:10209–23.

