# Supplementary material for: GPU-Accelerated Molecular Dynamics Simulation to Study Liquid Crystal Phase Transition Using Coarse-Grained Gay-Berne Anisotropic Potential
Source: PLoS One. 2016 Mar 17;11(3):e0151704. doi: 10.1371/journal.pone.0151704 (PMC4795799; doi:10.1371/journal.pone.0151704)
Supplement: S1 Fig — (DOC) [file pone.0151704.s001.doc]

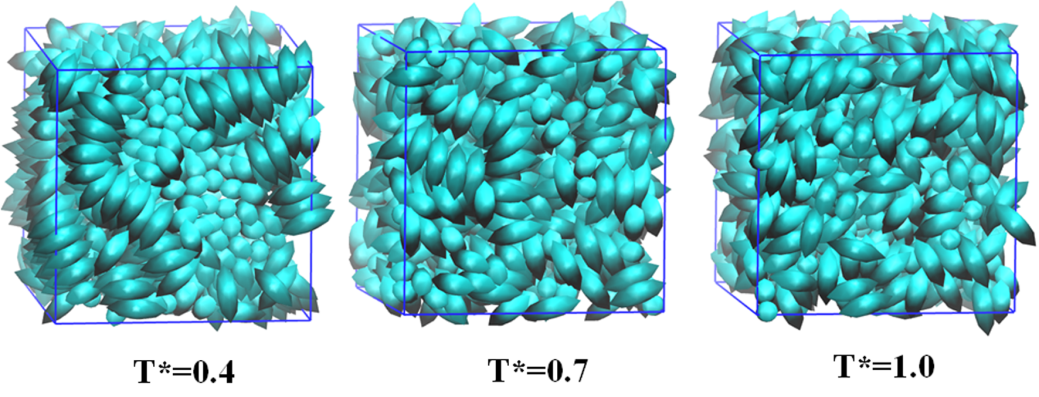


S1 Fig. Snapshots of typical phases for SCLCP without the presentation of backbones and spacers from low to high temperature, corresponding to multi-domain nematic to isotropic phase transition.
