# Supplementary material for: GPU-Accelerated Molecular Dynamics Simulation to Study Liquid Crystal Phase Transition Using Coarse-Grained Gay-Berne Anisotropic Potential
Source: PLoS One. 2016 Mar 17;11(3):e0151704. doi: 10.1371/journal.pone.0151704 (PMC4795799; doi:10.1371/journal.pone.0151704)
Supplement: S2 Fig — (DOC) [file pone.0151704.s002.doc]

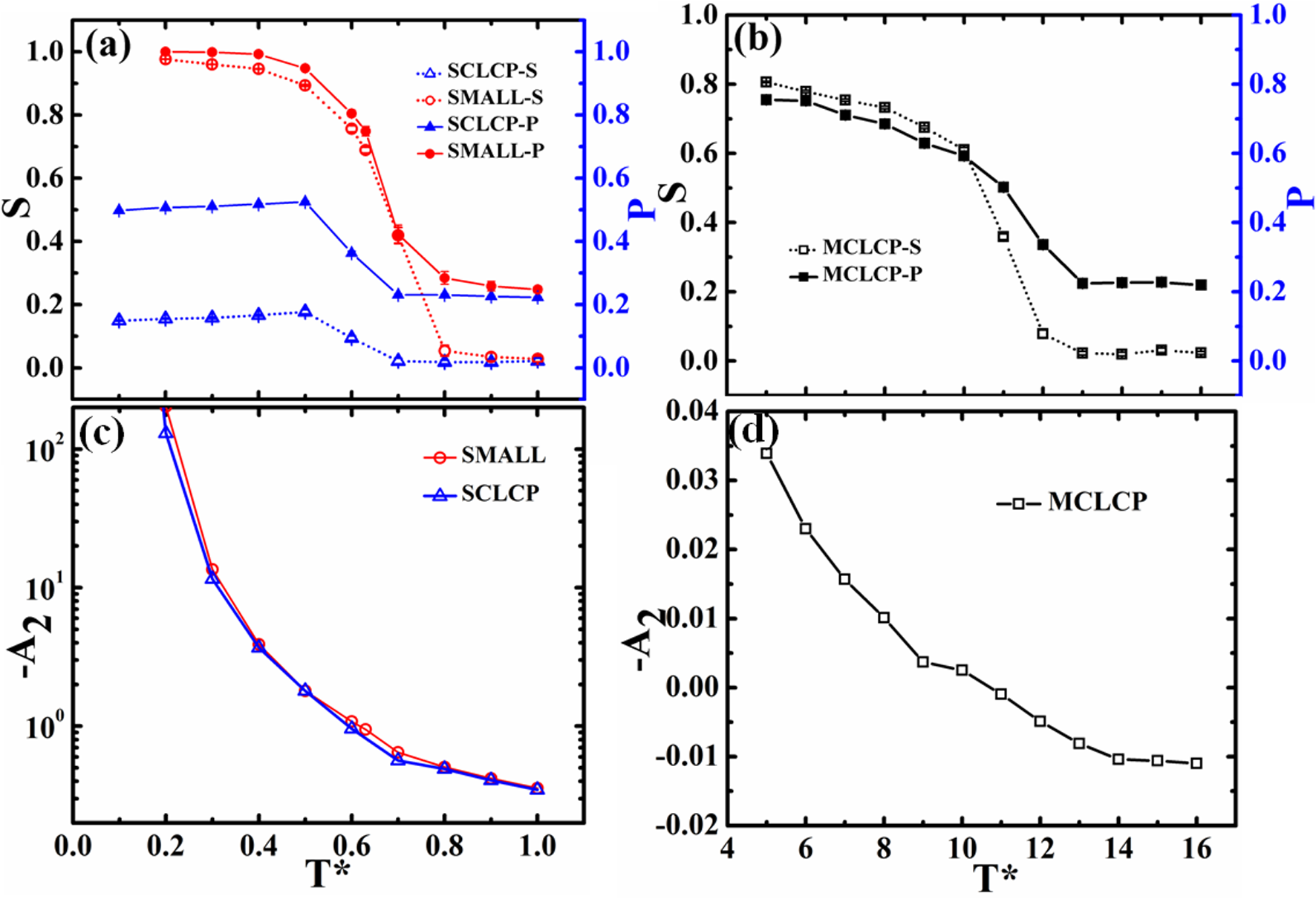


S2 Fig. The orientational order parameter *S*, the probability of the local orientation *P* (a, b), and the second virial coefficient *A*_2_ (c, d) as a function of temperature from large simulation box for mesogens in small molecular LC, SCLCP and MCLCP systems.
