# Supplementary material for: GPU-Accelerated Molecular Dynamics Simulation to Study Liquid Crystal Phase Transition Using Coarse-Grained Gay-Berne Anisotropic Potential
Source: PLoS One. 2016 Mar 17;11(3):e0151704. doi: 10.1371/journal.pone.0151704 (PMC4795799; doi:10.1371/journal.pone.0151704)
Supplement: S3 Fig — (DOC) [file pone.0151704.s003.doc]

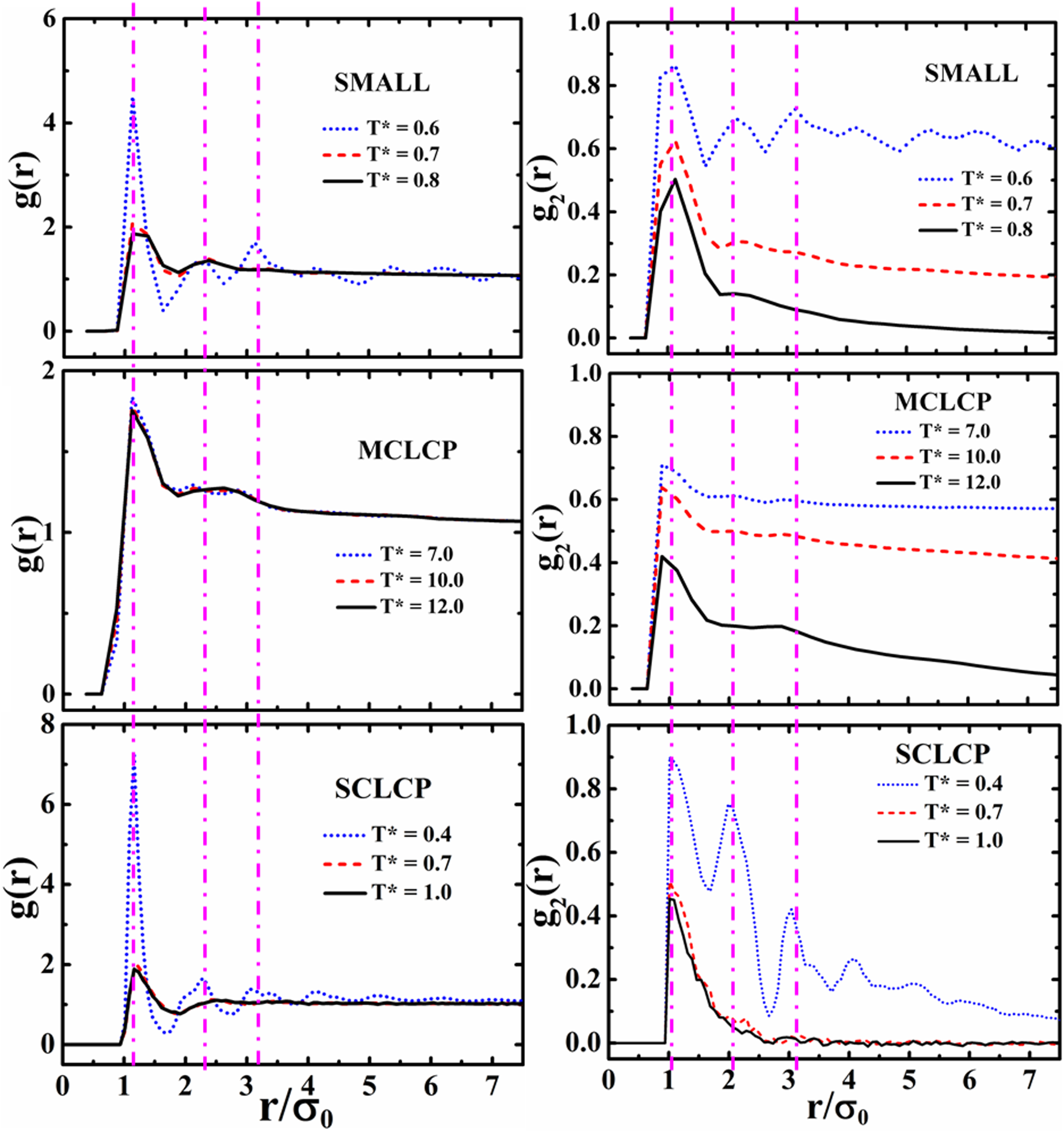


S3 Fig. The radial distribution function *g*(*r*) and the orientational correlation functions *g*_2_(*r*) at various temperatures from large simulation box for mesogens in small molecular LC, SCLCP and MCLCP systems.
